# Myotonic dystrophy type 1 in the COVID-19 era

**DOI:** 10.1007/s10072-023-06834-5

**Published:** 2023-05-08

**Authors:** Jelena Ilic Zivojinovic, Katarina Djurdjevic, Ivo Bozovic, Giovanni Meola, Marina Peric, Ana Azanjac Arsic, Ivana Basta, Vidosava Rakocevic-Stojanovic, Stojan Peric

**Affiliations:** 1 Institute of Hygiene and Medical Ecology, University of Belgrade - Faculty of Medicine, Belgrade, Serbia; 2 University Clinical Center of Serbia - Neurology Clinic, University of Belgrade - Faculty of Medicine, Belgrade, Serbia; 3 Department of Neurorehabilitation Sciences, Casa Di Cura Igea, Department of Biomedical Sciences for Health, University of Milan, Milan, Italy; 4 Mother and Child Health Care Institute “Dr. Vukan Cupic”, Belgrade, Serbia; 5 Department of Neurology, Faculty of Medical Sciences, University of Kragujevac, Kragujevac, Serbia; 6 Department for Neuromuscular Disorders, Neurology Clinic, University Clinical Centre of Serbia, Faculty of Medicine, University of Belgrade, 6, Dr Subotic Street, Belgrade, 11 000 Serbia

**Keywords:** Myotonic dystrophy type 1, COVID-19, SARS-CoV-2, Vaccination

## Abstract

**Introduction:**

Myotonic dystrophy type 1 (DM1) is the most prevalent muscular dystrophy in adults. People with DM1 might represent a high-risk population for respiratory infections, including COVID-19. Our aim was to evaluate the characteristics of COVID-19 infection and vaccination rate in DM1 patients.

**Methods:**

This cross-sectional cohort study included 89 patients from the Serbian registry for myotonic dystrophies. Mean age at testing was 48.4 ± 10.4 years with 41 (46.1%) male patients. Mean duration of the disease was 24.0 ± 10.3 years.

**Results:**

COVID-19 infection was reported by 36 (40.4%) DM1 patients. Around 14% of patients had a more severe form of COVID-19 requiring hospitalization. The severity of COVID-19 was in accordance with the duration of DM1. A severe form of COVID-19 was reported in 20.8% of patients who were not vaccinated against SARS-CoV-2 and in none of the vaccinated ones. The majority of 89 tested patients (66.3%) were vaccinated against SARS-CoV-2. About half of them (54.2%) received three doses and 35.6% two doses of vaccine. Mild adverse events after vaccination were recorded in 20.3% of patients.

**Conclusions:**

The percentage of DM1 patients who suffered from COVID-19 was like in general population, but with more severe forms in DM1, especially in patients with longer DM1 duration. The study indicated an overall favorable safety profile of COVID-19 vaccines among individuals with DM1 and its ability to protect them from severe COVID-19.

## Introduction

Myotonic dystrophy type 1 (DM1) is the most prevalent muscular dystrophy in adults, and it has a wide phenotypic spectrum [1]. It is a multisystemic disorder characterized by progressive muscle weakness with myotonia and degeneration of the heart, the brain, eyes, endocrine glands, and other organs [2–4]. Different metabolic syndromes, such as type 2 diabetes (T2D), are frequently a part of the DM1 phenotype [5, 6]. On the other hand, it has been estimated that DM1 patients are at higher risk of developing different types of cancers [7].

One of the worst disasters the world has faced in its recent history is the global epidemic of Coronavirus disease 2019 (COVID-19). The first outbreak of pneumonia related to the novel coronavirus was reported in Wuhan City, China, in December 2019. This novel coronavirus was typically presented with mild to severe respiratory disease in humans [8]. Over the next few months and years, growing concerns about the possibility that patients with chronic diseases may become more susceptible to COVID-19 were observed [9, 10]. About 10–30% of COVID-19 survivors may develop long-COVID or Post-COVID Syndrome (PCS), characterized by persistent symptoms (most commonly fatigue, dyspnea, and cognitive impairment) lasting 3 months or more after COVID-19 [11, 12].

As respiratory muscle weakness and cardiopulmonary dysfunction belong to the DM1 spectrum, people with these disorders might represent a high-risk population for respiratory infections, including COVID-19 [9, 10]. In addition, patients with DM1 often have glucose metabolism impairment. All in all, patients with DM1 seem to have three main risk factors for a severe form of COVID-19 [9, 13]. History of carcinoma is also a risk factor for developing severe COVID-19 [14], and DM1 patients are at risk of having carcinoma more frequently than the general population.

COVID-19 vaccination program started in February 2021 in Japan, with priority given to high-risk groups, including patients with muscular dystrophies (DM) [10]. The Muscular Dystrophy Association (MDA) has also recommended that patients with MD should avoid exposure to COVID-19 due to an increased risk of poorer infection outcome and a higher impact of the infection on patients’ daily life. In accordance with this, MDA has recommended vaccination against COVID-19 in patients with MDs [15]. Despite being recommended, safety and immunogenicity of COVID-19 vaccination in patients with DM1 are still unknown [10], and there is a concern among both patients and physicians about post-vaccination exacerbations and their potential side effects.

The aim of the research was to evaluate the characteristics of COVID-19 infection in a large cohort of DM1 patients, as well as the impact of this infection on the further course of the disease. We also analyzed the vaccination rate against severe acute respiratory syndrome coronavirus 2 (SARS-CoV-2) in DM1 patients and its potential influence on the course of the disease.

## Method

All data were collected from the “*Akhenaten*”, the Serbian registry for myotonic dystrophies, and conducted via telephone interviews by a survey specialist in the period from April to December 2022. Prior to filling in the questionnaire, all patients had provided written informed consent via email for participating in the study, and they had given permission to researchers for using the data.

**Fig. 1:**
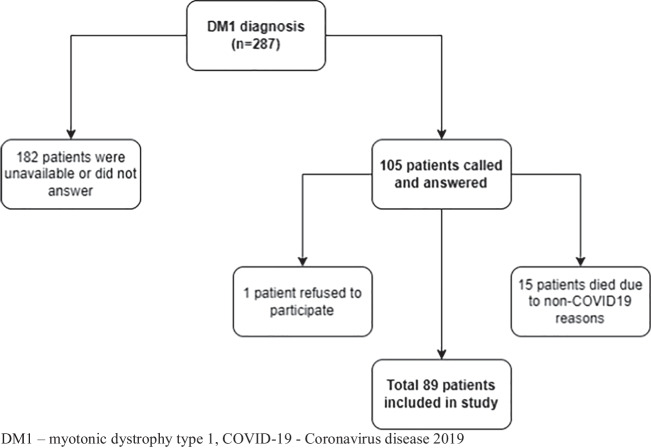
Flowchart representing included and excluded DM1 patients

This cross-sectional cohort study included patients listed in the Serbian registry for myotonic dystrophies. The clinical diagnosis of DM1 was confirmed in all patients using molecular genetic testing of the DMPK gene [16]. All DM2 patients, DM1 patients who were not genetically confirmed, as well as one female patient who had both DM1 and DM2, were excluded from this study. We identified 287 patients who fulfilled the initial criteria. The contacts of 182 patients were available in the Registry, and they were contacted by phone. Among them, 99 patients were unavailable or they had changed their contact details. Eighty-three patients were excluded after the minimum of three attempts to be reached on the phone; one patient refused to participate; and fifteen patients died before or during the COVID-19 pandemic; but neither of them died due to COVID-19. Thus, a total number of 89 patients were included in the final analysis (Fig. 1).

Sociodemographic data were taken from the Registry and additionally updated during the telephone interview (they included gender, age, marital status, education, disease onset, comorbidities, therapy, as well as disease symptoms). For testing purposes, a specifically designed questionnaire was made, and it consisted of five parts. The first part of the questionnaire referred to COVID-19 infection, while the second part collected data on vaccination status against SARS-CoV-2. All patients who were vaccinated against SARS-CoV-2 provided information about the type of vaccine and the number of doses they received. The third part of the questionnaire referred to patients’ mobility, as well as cardiovascular and pulmonary problems before and after COVID-19 infection. The fourth section was filled in only in case of a patient’s death. The information on patients who died was provided by their closest relatives or caregivers, and it included the time of death and cause of death. In the fifth part of the questionnaire, the patients were asked how they felt about their health and their illness compared to 2 years before (prior to the pandemic).

### Statistical analysis

The results are presented as proportions (%), means ± standard deviations depending on the data type. A comparison between groups was performed using chi square test, Mann-Whitney *U* test, or Student *t* test, depending on data type. Correlation was done with Spearman correlation coefficient. All *p* values below 0.05 were considered significant.

## Results

This research comprised 89 patients from the DM Registry (Fig. 1). The cause of death of 15 patients included cardiovascular problems (five patients), pulmonary problems (two patients), or other problems (gallbladder perforation, sepsis, suffocation by a foreign body, lung cancer, or other — eight patients).

The main sociodemographic and clinical data of examined DM1 patients are listed in Table 1. Mean age at testing was 48.4 ± 10.4 years, and there were 41 (46.1%) males. Mean duration of the disease was 24.0 ± 10.3 years. Most patients walked without assistance (68.5%), while about a fifth of all patients used unilateral support while walking (21.3%). Cardiovascular disorders before COVID-19 were recorded in 34 (38.2%) patients, and pulmonary problems in 35 (39.3%). These patients most often had conduction disorders and restrictive lung disease. Only one patient had both pulmonary thromboembolism (*PTE*) and *deep venous thrombosis* (*DVT*) in the period before COVID-19.

**Table 1:** Main sociodemographic and clinical features of examined DM1 patients

Demographic data	Number (%) or mean ± *SD*	*N*
Total	89 (100%)	
Sex — male	41 (46.1%)	
Age at the time of testing (years)	48.4 ± 10.4	
Duration of DM1 (years)	24.0 ± 10.3	
Cardiologic disorders before COVID-19	34 (38.2%)	
	- Arterial hypertension	11
	- Cardiac conduction defects	21
	- Arrhythmia	7
	- Cardiomyopathy	1
Pulmological disorders before COVID-19	35 (39.3%)	
	- Restriction	30
	- Obstruction	4
	- Non-invasive ventilation	1
DVT/PTE before COVID-19		1 (1.1%)
Walking ability before COVID-19	Without support	61 (68.5%)
	With the help of orthotics	1 (1.1%)
	With unilateral assistance	19 (21.3%)
	With bilateral assistance	4 (4.5%)
	Wheelchair, but can stand up	3 (3.4%)
	Wheelchair, but cannot stand up	1 (1.1%)

*SD* standard deviation; *DM1* myotonic dystrophy type 1; *DVT/PTE* deep venous thrombosis/pulmonary thromboembolism

### Characteristics of COVID-19 infection in DM1 patients

COVID-19 infection was verified in 36 (40.4%) DM1 patients. The majority of patients (72.2%) had mild forms of COVID-19, while 14% of patients had a more severe form of the disease, requiring hospitalization during the initial infection (Table 2). One (20.0%) out of five patients who had COVID-19 reinfection had a severe form of the disease. Only one patient had verified COVID-19 infection for the third time, and it was characterized as mild. Fifteen partners of our DM1 patients had COVID-19 infection and the majority (92.9%) of them suffered from a mild form. About a third of all patients (31.4%) got COVID-19 infection after vaccination, with the majority of them suffering from it after the third dose of the vaccine (54.5%). Mild post-COVID-19 sequelae after the first infection were recorded in only two (5.6%) patients, including the presence of headache, shortness of breath, significant fatigue, and chest tightness.

**Table 2:** Characteristics of the COVID-19 infection in DM1 patients

Feature		*N* (%) or mean ± *SD*
Total (*N*)		36 (40.4%)
COVID-19 infection once		31 (86.1%)
COVID-19 infection twice		4 (11.1%)
COVID-19 infection trice		1 (2.8%)
Severity of clinical presentation first time^a^		
1	Asymptomatic infection	5 (13.9%)
2	Mild clinical symptoms without hospitalization	26 (72.2%)
3	Hospitalization without O_2_ use	2 (5.6%)
4	Hospitalization with O_2_ use via mask or nasal cannula	2 (5.6%)
5	Non-invasive ventilation or *high-flow* O_2_	1 (2.7%)
Severity of clinical presentation second time		
1	Asymptomatic infection	1 (20.0%)
4	Hospitalization with O_2_ use via mask or nasal cannula	5 (80.0%)
Severity of clinical presentation third time		
1	Asymptomatic infection	1 (100.0%)
Partners with COVID-19 infection		14 (38.9%)
Severity of clinical presentation in partners during first infection		
1	Asymptomatic infection	4 (28.6%)
2	Mild clinical symptoms without hospitalization	9 (64.3%)
5	Non-invasive ventilation or *high-flow* O_2_	1 (7.1%)
Post-COVID sequelae for the first time*		2 (5.6%)
COVID infection after vaccination		11 (31.4%)
After the first dose		1 (9.1%)
After the second dose		4 (36.4%)
After the third dose		6 (54.5%)
Time between vaccination and COVID-19 (months)^b^		3.7 ± 2.7

^a^Categories 1 and 2 are considered milder form; categories 3–5 are considered more severe form

^b^Range is between 2 weeks and 7 months

*Headache, shortness of breath, significant fatigue, and chest tightness

The only sociodemographic and clinical factor associated with a more severe form of COVID-19 infection in DM1 patients was the dystrophy duration at the moment of infection (19.4 ± 8.8 years in patients with mild COVID-19 vs. 30.4 ± 14.7 years in patients with severe COVID-19, *p* = 0.02). It should be noted that 20.8% of patients who were not vaccinated against SARS-CoV-2 had a severe form of COVID-19 infection and none of the vaccinated ones (*p* = 0.10).

Among 36 DM1 patients who suffered from COVID-19, only one reported gait worsening after the infection — a patient walked without support before COVID-19 and started to use unilateral support after the disease. One patient reported a de novo cardiomyopathy, and no other cardiovascular complications due to COVID-19 were noted. Three (8.3%) DM1 patients reported novel respiratory symptoms, including prolonged cough, shortness of breath, and pleurodynia. No thromboembolic events were noticed either during COVID-19 infection or shortly after it.

When the self-rating scale for assessing a change in patients’ health status after COVID-19 was analyzed, almost half of the patients (48.3%) did not note any difference. However, 40 (44.9%) patients reported mild worsening, and 2 (2.2%) noted severe worsening of their state.

### Vaccination against SARS-CoV-2 in DM1 patients

The majority of tested patients (66.3%) were vaccinated against SARS-CoV-2 (Table 3). About half of the patients (54.2%) received three doses and 35.6% two doses of the vaccine. The majority of patients received Synopharm (54.2%), followed by Pfizer-BioNTech vaccine (28.8%). Adverse events after vaccination were recorded in 20.3% of patients, being most often local skin reactions (66.7%), flu-like symptoms (16.7%), or both (16.7%). When it comes to unvaccinated patients, two-thirds said they did not want to get vaccinated, while the rest of them were afraid of worsening the underlying disease. Neither of the patients reported that their general practitioner or neurologist advised they should not get vaccinated. We did not notice that any sociodemographic or clinical factor was associated with refusing vaccination.

**Table 3:** Vaccination against SARS-CoV-2 in patients with DM1

Vaccinated DM1 patients	*N* (%) or mean ± *SD*
Total (*N*)	59 (66.3%)
Reasons not to get vaccinated	
Does not want to	20 (66.7%)
Is afraid of DM1 worsening	10 (33.3%)
Number of doses	
One	2 (3.3%)
Two	21 (35.6%)
Three	32 (54.2%)
Four	4 (6.8%)
Vaccine type (manufacturer)	
Sinopharm	32 (54.2%)
Pfizer-BioNTech	17 (28.8%)
Sputnik V	3 (5.1%)
Astra Zeneca	1 (1.7%)
Combination — Pfizer-BioNTech and Sinopharm	5 (8.5%)
Combination — Pfizer-BioNTech and Sputnik V	1 (1.7%)
Adverse events	12 (20.3%)
Flu-like symptoms	2 (16.7%)
Local reaction	8 (66.7%)
Flu-like symptoms and local reaction	1 (8.3%)
Vomiting	1 (8.3%)
Dose after which adverse events occurred	
First	8 (66.7%)
Third	2 (16.7%)
First, second and third	2 (16.7%)

## Discussion

The results of our study show that 40% of DM1 patients had COVID-19 infection, which is slightly more compared to the general population of the Republic of Serbia (around 37% on February 15th, 2023) [17]. To the best of our knowledge, no data on COVID-19 prevalence in DM1 patients were available from other populations.

The percentage of DM1 patients that required hospitalization due to COVID-19 was 14% during the first infection in comparison with only 7% in their partners, which suggests that there is a predisposition of DM1 to more severe disease forms [9]. In accordance with this is the association between COVID-19 severity and duration of DM1 disease at the time of infection. Epidemiological studies that have been carried out recently, as well as the identification of the main risk factors for increased severity/mortality of the disease, are of great importance for improving the treatment of the patients. Our results suggest that DM1 patients with longer disease duration are at more pronounced risk of developing a severe form of COVID-19. A survey conducted among general population in Japan showed that risk factors for severity/mortality of COVID-19 were similar across pandemic waves, including older age, male sex, history of malignancy, congestive heart failure, and chronic obstructive pulmonary disease [18]. We were not able to confirm that any of these factors was a predictor of a worse outcome of COVID-19 in DM1 patients. In a recent paper, Mazzitelli et al. reported two DM1 patients with COVID-19 and found only five additional cases in literature. The majority of them had lethal outcome, while severity of DM1, obesity, and cardiovascular diseases were common in deceased patients [19].

In individual cases, COVID-19 was capable of worsening DM1 disease, having the effect on patient’s walking ability and their cardiovascular and pulmonary status. When self-estimated, a worsening was reported by almost half of DM1 patients. This worsening may be related not only to patients’ physical problems, but also to their emotional and social status that was certainly affected during the pandemic [20].

Most of our patients (66%) were vaccinated against SARS-CoV-2, much more compared to the general population of the Republic of Serbia (49% on February 15th, 2022) [21]. This suggests that DM1 patients were well educated and that they understood the risk of COVID-19. The most frequently reported reason for not getting vaccinated was fear of worsening the underlying disease. This means that further educaton is needed in DM1 patients. Avoiding vaccination may be related to their cognitive defects and personality traits [22, 23]. A recent survey conducted in Serbia in general population lists five reasons for the population's hesitancy about getting vaccinated. They refer to the side effects of the vaccine, concerns over their effectiveness, concern over insufficiently tested vaccines, distrust of authorities, and conspiracy theories [24]. Some of these factors may also be related to DM1.

More than half of the patients (54%) received the Sinopharm vaccine, followed by Pfizer-BionTech (29%). This corresponds to the data from general population of Serbia from mid-May 2022, where the highest coverage of adults with the primary vaccine series was by Sinopharm (58%) and Pfizer/BioNTech (28%), followed by Sputnik V (9.5%), AstraZeneca (4.1%), and Moderna (0.01%) [25]. We did not note any differences regarding the efficacy of the vaccine and its safety, although our cohort is too small to make any conclusions. The adverse effects after vaccination occurred in one fifth of our DM1 patients (20%), which was approximately twice as common as that previously reported in the general population [26], but less frequent than in our patients with myasthenia gravis (36%) [14]. Patients most frequently reported the occurrence of a local reaction (67%), which is similar to the results of a previous study [10]. No severe adverse events were noted. Another study conducted in the Serbian general population showed that the majority of adverse reactions to all four vaccines were local reactions at the injection site, which lasted for several days, and included fever, pain, swelling, and redness, followed by systemic reactions such as fever, pain in the muscles, joint pain, tremors, weakness, headache, and nausea [26]. There were no reports of worsening of DM1 symptoms and signs in our patients, including muscle strength and their cardiovascular and pulmonary status after vaccination. Moreover, our vaccinated DM1 patients might develop COVID-19, but none of them had a severe form of COVID-19. These data suggest that vaccines against COVID-19 are safe and effective in DM1.

The main limitation of our study is the fact that we obtained data via telephone call at one moment, which may lead to recall bias. However, despite the limitations, this cohort includes a large number of patients and provides significant data regarding COVID-19 infection and COVID-19 vaccination in DM1 patients.

## Conclusions

The percentage of COVID-19 infection was similar in DM1 patients and in the general population, but with more severe cases of COVID-19 in DM1, especially in those patients with longer DM1 duration. COVID-19 may have an impact on physical and psychological health of DM1 patients. The study indicated an overall favorable safety profile of COVID-19 vaccines among individuals with DM1 with their protective effect from severe COVID-19.

## Data Availability

The data that support the findings of this study are available on request from the corresponding author. The data are not publicly available due to privacy and ethical restrictions.
